# Enhancing high-performance concrete sustainability: integration of waste tire rubber for innovation

**DOI:** 10.1038/s41598-024-55485-9

**Published:** 2024-02-26

**Authors:** Dhipan Aravind Singaravel, Pavalan Veerapandian, Silambarasan Rajendran, Ratchagaraja Dhairiyasamy

**Affiliations:** 1 Department of Civil Engineering, Annapoorana Engineering College (Autonomous), Salem, Tamil Nadu India; 2 Department of Civil Engineering, Sengunthar Engineering College (Autonomous), Thiruchengode, India; 3 Department of Mechanical Engineering, Annapoorana Engineering College, (Autonomous), Salem, Tamil Nadu India; 4 Department of Mechanical Engineering, College of Engineering and Technology, Aksum University, Aksum, Tigray Ethiopia

**Keywords:** High-performance concrete, Waste tire rubber, Aggregate, Sand replacement, Mechanical properties, Dynamic properties, Environmental impact, Energy science and technology

## Abstract

This study extensively explored the impact of integrating waste tire rubber into high-performance concrete (HPC) by substituting natural sand. Different fractions of rubber particles—5%, 10%, and 15% replacements of the fine aggregate—were rigorously investigated. Properties from fresh to hardened concrete were assessed, including compressive and tensile strength, modulus of elasticity, workability, and damping coefficient. Replacing up to 10% of sand with 0.6 mm rubber particles showed minimal strength compromise compared to standard HPC. However, at a 15% replacement rate, a noticeable decline in strength became evident, highlighting an optimal threshold for inclusion. Additionally, rubber incorporation notably enhanced concrete ductility and damping, marking a substantial improvement in dynamic properties. Efforts to offset strength reduction through increased fines content and mineral admixture could not counteract the decline at the 15% replacement level, suggesting limitations in compensatory measures. Methodological refinements enhanced data accuracy, including capping and surface treatments during compression testing. The study underlined the viability of controlled rubber substitution for bolstering HPC's dynamic attributes. Despite strength reductions at higher replacement rates, controlled waste tire rubber integration proves promising for enhancing HPC's dynamics without compromising structural integrity, advocating its suitability across diverse construction applications.

## Introduction

Concrete is the most widely used construction material globally, with annual consumption estimated at 11 billion metric tons. However, standard concrete production has major environmental impacts due to the energy and emissions associated with cement manufacturing and the extraction of virgin aggregates. There is growing interest in making concrete more sustainable by using waste materials as replacements for conventional ingredients. Scrap tires represent a major solid waste challenge, with over 1 billion tires discarded annually worldwide. Landfilling and stockpiling of waste tires cause serious environmental and health hazards. Therefore, reusing scrap tire rubber as an aggregate substitute in concrete can tackle two pressing issues simultaneously.

Crumb rubber from waste tires has been studied as a replacement for natural fine aggregates in concrete for decades. Research shows that adding tire rubber can positively enhance properties like toughness, impact/fatigue resistance, vibration damping, and thermal insulation. However, a key challenge is that increasing rubber content reduces compressive and tensile strength. Most studies conclude that replacements beyond 10–15% compromise strength excessively for structural concrete. High-performance concrete (HPC) is an advanced formulation that utilizes optimized gradation, reduced porosity, and mineral admixtures to achieve higher strength. The effects of waste tire rubber on HPC have not been widely investigated.

Abdolpour et al. studied spent equilibrium catalysts and steel fibers from scrap tires in ultra-high-performance concrete. They found 15% lower CO_2_ emissions and 6% lower cost with catalyst replacement but limited strength change^1^. Agrawal et al. investigated pretreating rubber fibers from discarded tires to improve rubberized concrete properties. Treating with acids or alkalis enhanced strength versus untreated rubber, with 10% pretreated rubber maintaining strength while improving ductility^2^. Al-Osta et al. incorporated recycled rubber and polyethylene into concrete, finding 40% lower thermal conductivity but over 80% lower strength^3^. Thermal resistance improvements reduced estimated energy use, emissions, and costs over 30 years. Al-Tarbi et al. produced concrete blocks using recycled rubber, finding blocks met strength requirements with polyethylene but not rubber. New block configurations significantly improved thermal resistance over commercial blocks^4^. Ashokan, Jaganathan et al. performed a life cycle assessment of concrete block manufacturing, determining sustainable strategies like industrial byproducts to reduce environmental impact^5^. Ashokan, Rajendran et al. added nano-silica to steel microfiber concrete, finding 1% nano-silica and 2% microfibers optimal for strength and toughness. This shows potential for reducing construction waste^6^.

El-Seidy et al. investigated alkali-activated materials with recycled unplasticized polyvinyl chloride aggregates for sand replacement, showing enhanced thermal resistivity and chloride penetration resistance^7,8^. Sambucci and Valente studied total sand replacement by tire rubber in concrete for lightweight paving blocks, finding suitable performance and eco-friendly features^9^. Sambucci and Valente analyzed the effect of rubber particle size on 3D printable cement mortars, showing an influence on print quality, microstructure, and properties^10^. Sambucci and Valente optimized the thermal insulation of 3D printable hollow bricks with fractal inner cavities using FEM, increasing thermal resistance^11^. Valente et al. compared alkali-activated and Portland cement materials with recycled tire rubber, revealing greater environmental benefits but higher costs for geopolymers^12^. Valente et al. incorporated waste tire rubber in alkali-activated materials for 3D printing, demonstrating good printing, mechanical, thermal, and acoustic properties^13^.

Celestino added UV-treated recycled rubber to the mortar, finding strength decreased, but deformability increased with higher rubber content. Treating rubber improved properties over raw rubber^14^. Mixti et al. incorporated recycled tire materials into geopolymer mortars, finding reduced environmental impact and improved durability, though slightly reduced strength. Optimizing mixture design saved over 50% of emissions. Sambucci et al. conducted a life cycle assessment of 3D printed and cast cementitious materials with ground tire rubber, defining an index to quantify overall performance^15^. Flores Medina et al. studied rubberized concrete durability, finding that larger rubber particles improved freeze–thaw and acid resistance. UPV effectively assessed degradation, while fibers improved cohesion^16^. Gorde reviewed using treated crumb rubber as a fine aggregate in concrete, finding rubber requires treatment to improve concrete properties and bond with the cement matrix^17^. Juveria et al. combined recycled concrete aggregate and tire rubber stabilized with slag, finding that the mixtures met the requirements for pavement base layers. Stabilization allowed higher waste material use^18^. Karimi et al. added varying sizes and amounts of recycled rubber to concrete, determining 4% coarse rubber-optimized strength and cracking resistance. Fine rubber decreased properties^19^. Karunarathna et al. found rubberized concrete showed increased ductility and toughness under impact, especially with larger rubber particles. Numerical modeling effectively predicted the effect^20^. Liu et al. found that adding rubber aggregate to self-compacting concrete improved dynamic compressive behavior and energy absorption under multiple impacts^20^. Los Santos-Ortega et al. performed a life cycle assessment of mortar with crumb rubber addition. Up to 40% rubber reduced emissions and fossil fuel use, though transport distance affects viability^21^. Ma et al. analyzed the dynamic compression of slag-activated mortar with crumb rubber addition. Increasing rubber content decreased strength, attributed to poor matrix bonding. An optimal mix improved early strength^22^. Mei et al. developed a rubber-sand concrete with waste tire rubber for seismic isolation. Over 30% rubber or under 50% cement allowed adequate deformation capacity. Dynamic properties depended on composition^23^. Miah et al. found waste tire fine aggregate replacement of up to 20% maintained adequate strength in concrete. Higher replacement increased porosity and reduced strength, modulus, and shrinkage^24^.

Ren et al. reviewed crumb rubber as a fine aggregate in concrete, finding a poor bond with cement paste causes workability and strength reductions^25^. Shahjalal et al. reviewed fiber addition to improve the properties of concrete with recycled aggregate and crumb rubber. Fibers compensated for strength reductions from rubber and improved mechanical performance^26^. Thakare et al. added waste tire fibers and bacteria to self-compacting mortar, finding that using fibers as bacteria carriers enhanced strength and self-healing versus direct bacteria addition^27^. Tran et al. reviewed surface treatments for recycled tire rubber in cementitious materials. Chemical and physical treatments improve interfacial properties and recover lost strength^28^. Ul Islam et al. reviewed combining fibers and recycled tire rubber in concrete. Fibers compensate for reduced strength from rubber, improving static and dynamic mechanical properties^29^. Valente et al. 3D printed alkali-activated rubberized concrete, finding improved thermal and acoustic insulation over Portland-based composites with properly graded rubber inclusion^15^. Xiao et al. reviewed recycling approaches for waste tires, including devulcanization techniques to improve recovered rubber usability. Energy and chemical recovery methods are also discussed^30^. Xu et al. added steel fibers and porcelain waste to crumb rubber concrete, finding optimized strength with 25% porcelain and sufficient fiber addition to control cracking^31^. Zhang et al. partially replaced polyvinyl alcohol fibers with waste tire steel fibers in engineered cementitious composites. This improved properties while reducing cost and emissions^32^. Zhuo et al. incorporated recycled rubber and steel fibers into high-strength concrete, maintaining ultra-high performance with optimized rubber and fiber amounts^33^. Zia et al. added a hybrid of industrial and tire steel fibers to concrete, finding significant improvements to sustainability and mechanical properties versus plain concrete^34^.

The integration of waste tire rubber into concrete has undergone extensive research, but this work presents a unique perspective by concentrating on high-performance concrete (HPC), introducing several distinctive elements to the discourse. Previous studies predominantly explored conventional strength concrete, neglecting the distinct performance demands of HPC, resulting in limited research on rubberized HPC. This paper adopts a systematic methodology to analyze how rubber impacts HPC properties, manipulating replacement percentages and particle sizes within the original fine aggregate size range. Emphasizing the quantification of tradeoffs between strength, ductility, and vibration damping in controlled rubber substitution within HPC could benefit applications in constructing resilient structures resistant to impact and earthquakes. Moreover, methodological enhancements, such as implementing capping during compression testing, elevate data accuracy compared to earlier studies. This study offers fresh insights beyond existing literature by comprehensively evaluating fresh, hardened, and dynamic properties specific to rubberized HPC. Essentially, this research diverges from the norm by centering on high-performance concrete, employing a systematic approach to gauge effects on HPC properties, exploring tradeoffs between various factors, refining methods for enhanced data precision, and comprehensively evaluating rubberized HPC properties.

This study aimed to experimentally evaluate the influences of waste tire rubber as a sand substitute on the structural performance, workability, and dynamic characteristics of HPC. The novelty lies in focusing specifically on HPC rather than normal concrete and utilizing rubber particles in the size range corresponding to the original fine aggregate as a direct replacement. Systematically varying the rubber content would permit quantifying the tradeoffs between strength, ductility, and vibration damping. The results can potentially demonstrate the feasibility of waste tire rubber to improve the long-term durability and service life of HPC through enhanced energy absorption while retaining adequate strength for structural applications.

## Materials and methods

The high-performance concrete (HPC) mixtures utilized Portland cement, drinkable water, crushed granite as coarse aggregate, river sand as fine aggregate, silica fume, Superplasticizer, and waste tire rubber. The cement was Portland type II, commonly used for conventional concrete construction in India. The coarse aggregate was locally sourced granite with sizes between 9.5 and 25 mm. The fine aggregate was natural river sand with a fineness modulus of 2.66 and sizes between 0.15 and 4.8 mm. The silica fume was a supplementary cementitious material to improve particle packing and reduce porosity. A polycarboxylate-based superplasticizer allowed for the reduction of the water content while maintaining adequate workability. The waste tire rubber was obtained from a tire retreading company and separated into different-size fractions. The rubber particles passing a 0.6 mm sieve, retaining on a 1.19 mm sieve, and retaining on a 2.36 mm sieve were individually utilized as sand replacements. The rubber content replaced 5%, 10%, or 15% of the fine aggregate by volume. The HPC mixture proportions were designed to achieve a 28-day compressive strength exceeding 40 MPa. The optimal proportions were determined to be 1 part cement, 1.15 parts sand, 3.15 parts coarse aggregate, and a water/cement ratio of 0.32. Mass relative incorporated 8% silica fume and 0.08% superplasticizer into the cement. The rubber aggregate was substituted for an equivalent volume of sand in the reference mixture. The concrete was produced by mixing in a laboratory drum mixer. The coarse aggregate, half the mixing water, and sand were first added. After 2 min, the cement, silica fume, remaining water, and Superplasticizer were incorporated. The rubber was included with the fine aggregate for the modified mixtures. The fresh HPC was evaluated for slump, temperature, air content, and unit weight per ASTM standards. The hardened concrete was tested at 7, 28, and 56 days for compressive strength, splitting tensile strength, modulus of elasticity, and damping ratio. Scanning electron microscopy and image analysis characterized the microstructure and interfacial transition zone. The primary binder, Type II Portland cement (CP II–Z–32), adheres to ABNT NBR 11578:1991 standards. It features specific characteristics detailed in Table 1, including chemical parameters such as Loss to fire, Magnesium Oxide, Sulfur Trioxide, and Carbonic acid. Physical and mechanical attributes like Specific area, Setting start time, and Compressive strength at different intervals were also observed.

**Table 1:** Characteristics of cement.

Chemical parameters	
Loss to fire	< 6.5 (% mass)
Magnesium oxide	< 6.5 (% mass)
Sulfur trioxide	< 4.0 (% mass)
Carbonic acid	< 5.0 (% mass)
Physical and mechanical parameters	
Specific area	> 260 m^2^/kg
Setting start time	> 1 h
Compressive strength	
At 3 days	> 10.0 Mpa
At 7 days	> 20.0 Mpa
At 28 days	> 32.0 Mpa

**Figure 1:**
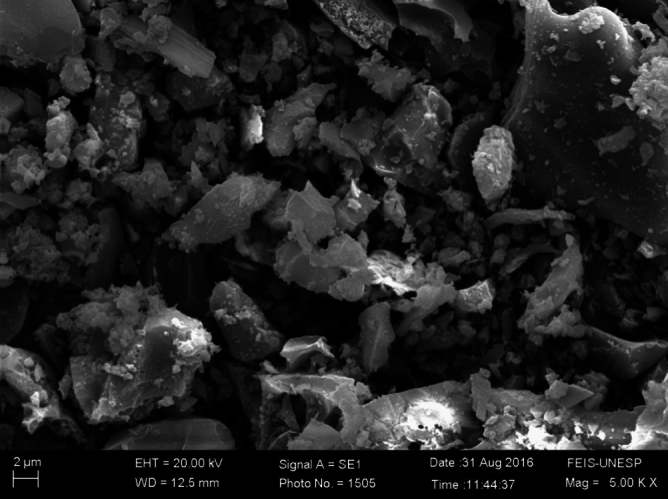
Particles of rice husk ash analyzed under a microscope.

The aggregates comprised zero gravel sourced locally, sand from a nearby port, and rubber waste from a local company. These underwent a drying process outdoors to attain near-zero humidity before storage for concrete production. The specific mass of these aggregates is presented, while Fig. 1 illustrates the particles of a sample of rice husk ash, analyzed in the electron microscope, where grains can be observed, mostly in the particle size range of 2 to 10 μm.

The concrete in this study was enhanced with key additives from reputable suppliers. Silica Fume, sourced from Astrra Chemicals in Mumbai, India, boasted an impressive 95% Silica (SiO_2_) content, a specific surface area ranging from 15,000 to 30,000 m^2^/kg, and a 2.20 g/cm^3^ density. Functioning as a crucial filler, its primary role was to enhance particle packing density and reduce porosity in the concrete. Rice Husk Ash, supplied by RHA Tech in Chennai, India, featured a Silica (SiO_2_) content between 85 and 95%, a specific surface area of 15,000–25,000 m^2^/kg, and a 2.05 g/cm^3^ density. Acting as a supplementary cementitious material contributed to the study's objectives. The Superplasticizer, Conplast SP430 from Fosroc Chemicals in Mumbai, India, was a sulfonated naphthalene formaldehyde-based brown liquid with a specific gravity of 1.2 and nil chloride content. Its primary role as a high-range water reducer was to disperse fine particles and decrease water demand, enhancing overall workability. These additives, with their distinct characteristics, played crucial roles in achieving the desired properties in the concrete mixtures studied.

The rubber particles employed in the study were sourced from a local tire retreading company as crumb rubber derived from scrap tires. These particles exhibited an apparent particle density of 1.15 g/cm^3^, significantly lower than the natural sand's 2.65 g/cm^3^. Micrograph analysis revealed a rough, porous surface morphology with fibrous textures originating from the original tire material, showcasing an irregular angular shape. The rubber's chemical composition included approximately 50–60% styrene-butadiene rubber, 20–30% natural rubber, 15–20% carbon black, 5–10% extender oils, and 5% other tire manufacturing chemicals. Particle size distribution, categorized into fine, medium, and coarse fractions, showcased varying characteristics, with the coarse fraction retaining more of the original shredded tire shape. The rubber particles exhibited a lower specific surface area than sand, varying absorption rates, and notably, a 6% water absorption in 24 h, contrasting with the 1% absorption in the sand. These unique properties, including lower density, angular shape, larger size, absorbent surface, and fibrous texture, contributed to the observed impacts on fresh and hardened concrete properties when utilized as a substitute for sand in the study. Rubber waste was segregated into various size ranges, showcasing the average tire composition. Comprehensive materials characterization tests have been diligently conducted, and their detailed results are provided in the supplementary file for thorough examination.

**Figure 2:**
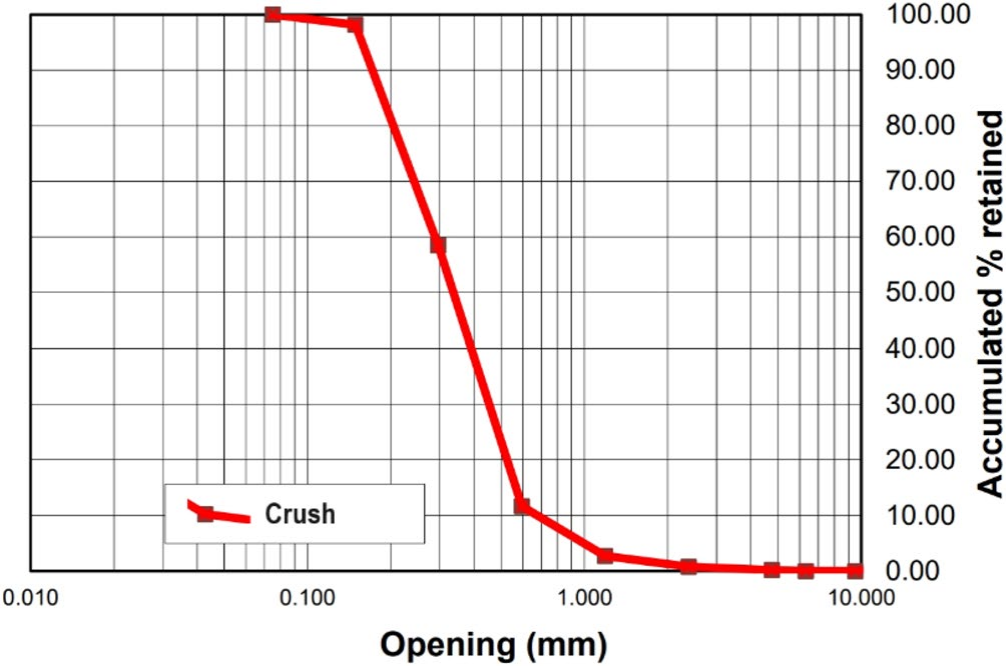
Particle size curve of the sand.

**Figure 3:**
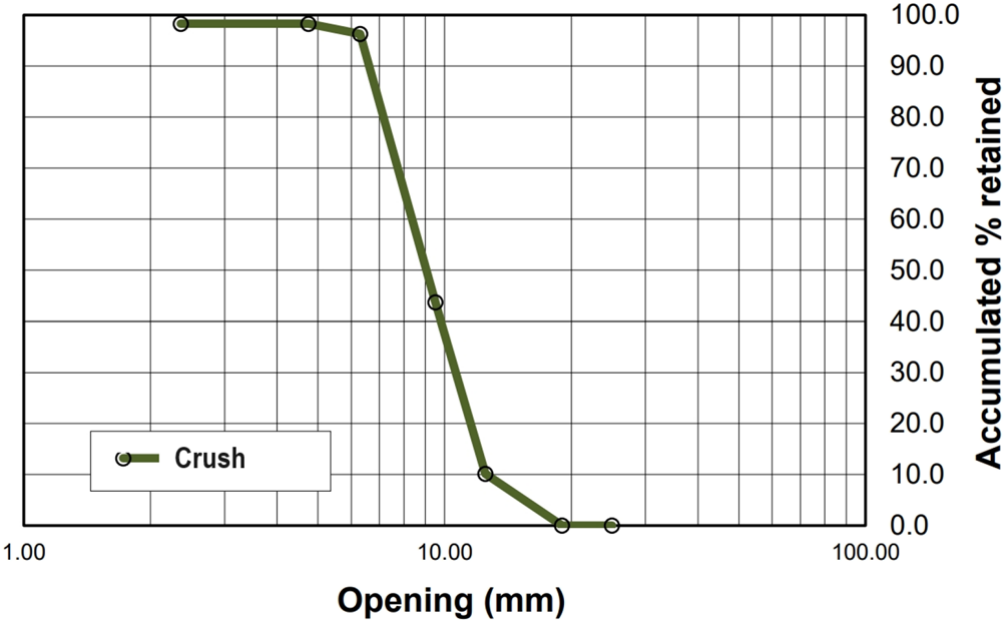
Particle size curve of zero gravel.

Figure 2 shows the granulometric curve resulting from the test carried out for sand, and Fig. 3 shows the granulometric curve resulting from the test carried out for zero crushed stone.

**Figure 4:**
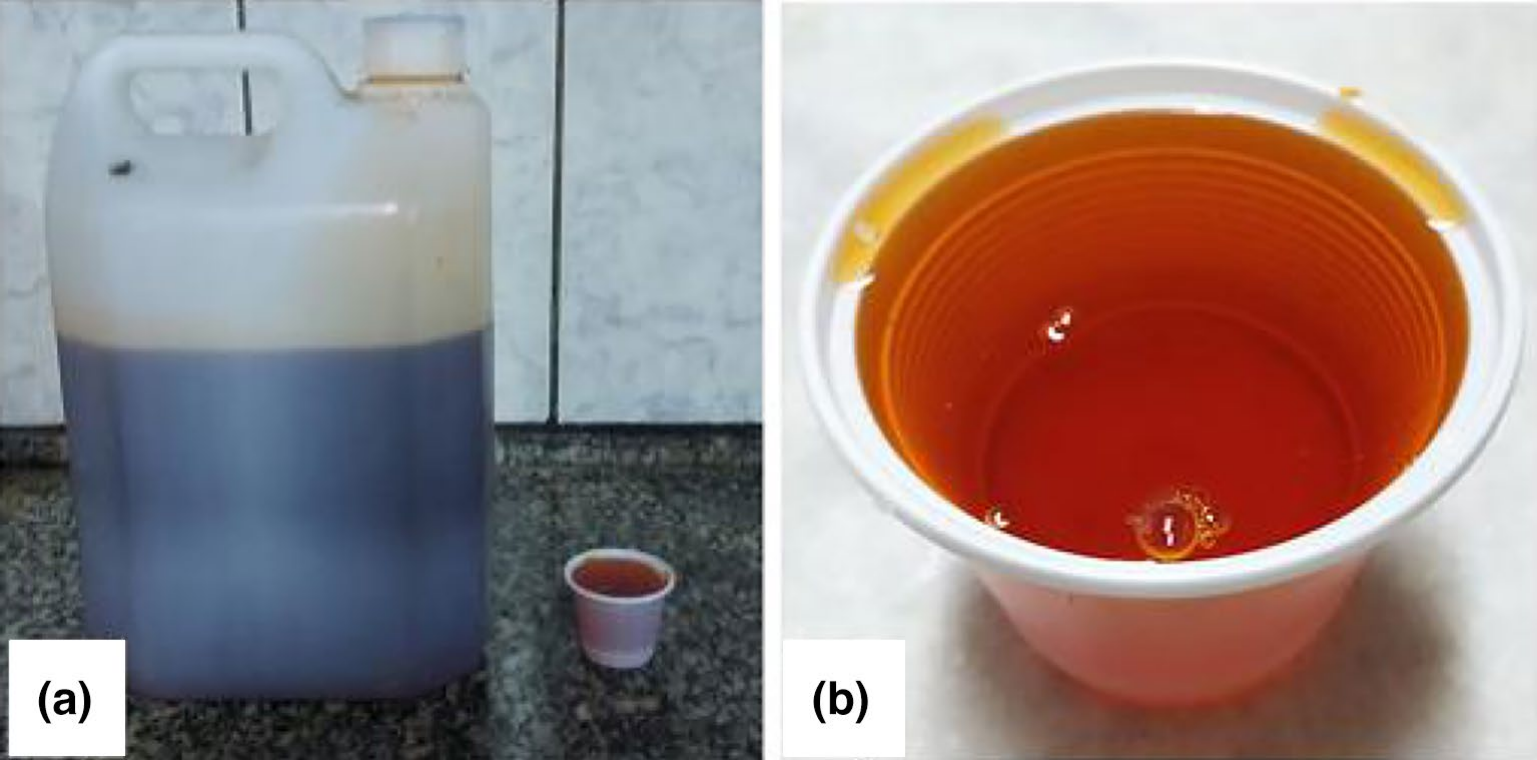
(**a**) Superplasticizer sample; (**b**) liquid, in detail.

**Figure 5:**
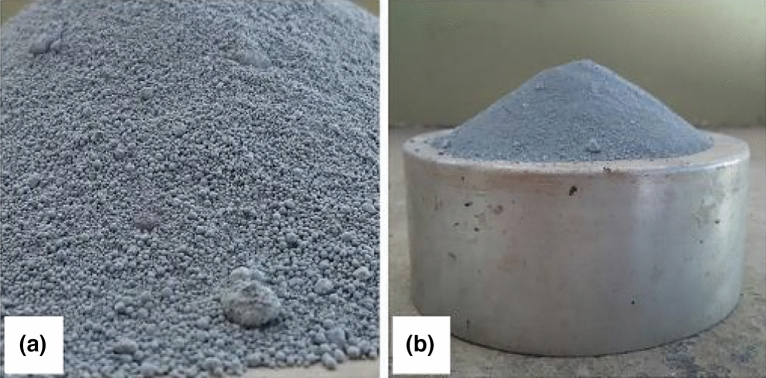
(**a**) Silica fume; (**b**) lack of cohesiveness.

**Figure 6:**
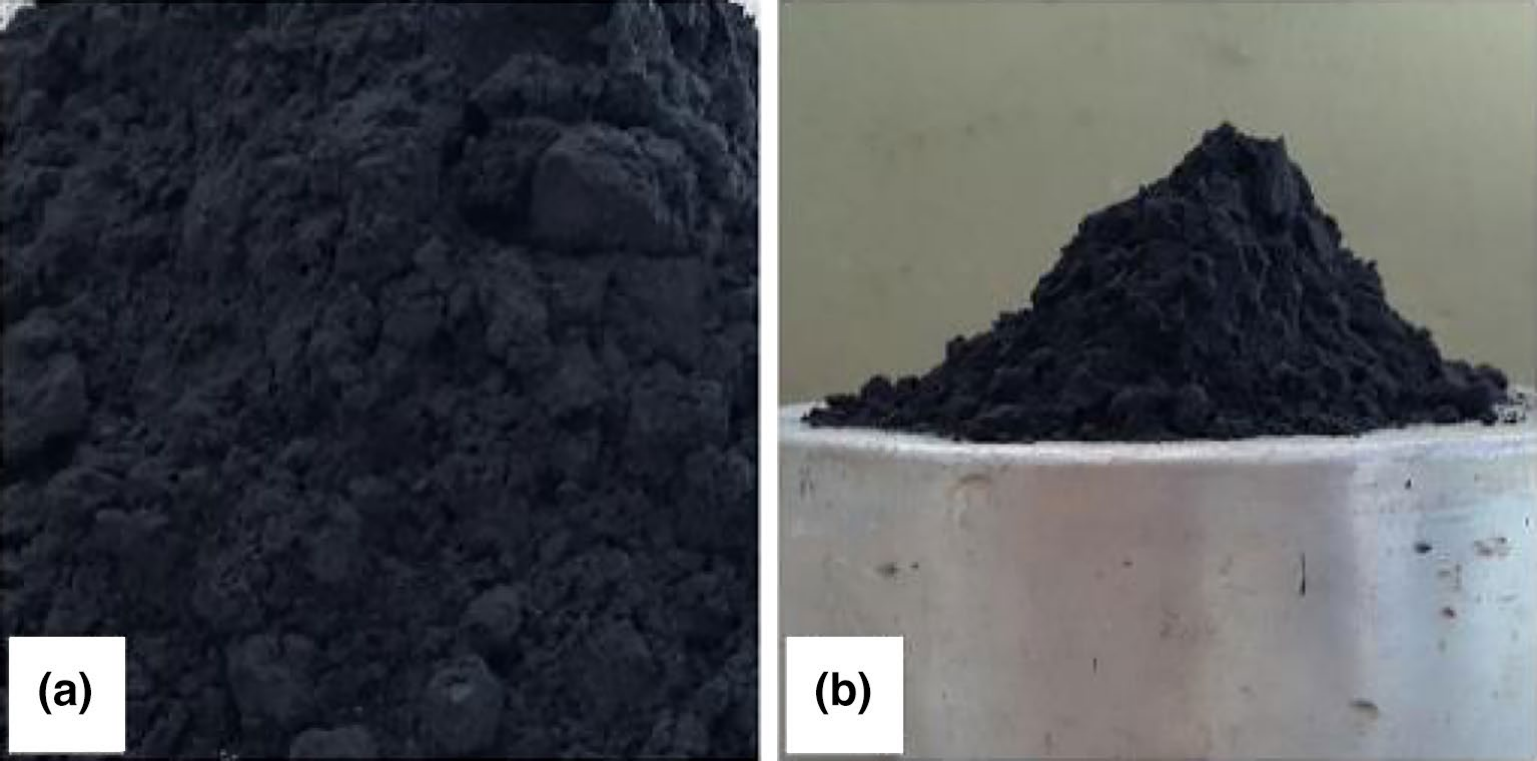
(**a**) Rice husk ash; (**b**) cohesive aspect.

The additives included a superplasticizer (Modified polycarboxylate solution) enhancing concrete workability, silica fume for smaller voids, and rice husk ash serving a similar role. Technical information about these additives is presented in Figs. 4, 5, and 6, illustrating their properties, appearance, and microscopic analysis.

## Experimental setup and procedure

The rubber aggregate, obtained from shredded scrap tires, was sorted into three-size fractions using sieves (0.6 mm, 1.19 mm, and 2.36 mm). Particle characteristics, such as size distribution, specific gravity, and water absorption, were assessed per ASTM C136 and ASTM C128. HPC mixtures were created in a rotary drum mixer, combining dry-blended aggregates with cement, silica fume, 80% water, and Superplasticizer. After an initial 2-min mix, additional water was added for the target slump; the total mixing time was fixed at 15 min. Mixtures were tested for slump (ASTM C143), temperature (ASTM C1064), unit weight (ASTM C138), and air content (ASTM C231). Each mixture produced eighteen 10 × 20 cm cylindrical specimens, three 15 × 15 × 50 cm prisms, and three 10 × 10 × 50 cm prisms compacted on a vibrating table. After casting, specimens were covered with plastic sheeting and moist cured for 28 days at 23 ± 2 °C and > 95% relative humidity. Cylindrical samples were tested for compressive strength (ASTM C39) and splitting tensile strength (ASTM C496) at designated ages. Dynamic modulus of elasticity was measured following ASTM C215. 15 × 15 × 50 cm prisms underwent four-point bending for flexural strength (ASTM C1609). The damping ratio was assessed by impacting 10 × 10 × 50 cm prisms and measuring the decaying vibration response. Scanning electron microscopy and image analysis examined microstructure and air void distribution.

The experimental study unfolded in six phases (I to VI), each with specific objectives. Phase I aimed to identify a mixture with compressive strength over 40 MPa for Concrete with Aggregate Discarded (CAD). Due to equipment issues at 28 days, evaluations were done at 7 days. Despite wet chamber malfunctions, Phase II established the Phase I mixture as a reference. Variations, such as replacing 5% of sand with rubber, led to the next Phase. Phase III assessed the impact of 5%, 10%, and 15% rubber residue on compressive strength, selecting the mix with a significant strength decrease for further study. Phase IV examined surface treatments on the chosen mix, compared with Phase I. Phase V reproduced the chosen Mix from Phase I and Phase III, introducing an alternative mix with increased rice husk ash content. Standardized production and testing processes ensured comprehensive comparisons. Lastly, Phase VI analyzed damping coefficients of concretes from Phases III and V. Consistent curing and surface treatments ensured reliable results throughout the study. Table 2 provides a summarized nomenclature of mixes in the initial phases for reference.

**Table 2:** Mixes used in the work.

Phase	Nomenclature	Description of mixes
Phase I	T1	Mix of 1:2:3
	T2	Mix of 1:3:3
	T3	Mix of 1:2:2
	T4 or T_REFERENCE1_	Mix of 1:1,15:3,15
Phase II	T_REFERENCE2_	Reference mix, identical to T4
	Tb less than 0.6 mm	Rubber with a grain size of less than 0.6 mm
	Tb equal to0.6 mm	Rubber with grain size between 0.6 mm and 1.19 mm
	TB greater than 0.6 mm	Rubber with grain size between 1.19 mm and 2.39 mm
Phase III	T_REFERENCE3_	Reference mix, identical to T4
	T15%	Mix with 15% waste
	T10% OFF	Mix with 10% waste
	T5%	Mix with 5% waste
Phase IV	T_REFERENCE4_	Reference mix, identical to T4
	T15%	Mix with 15% waste
Phase V	T_REFERENCE5_	Reference mix, identical to T4
	T15%	Mix with 15% waste
	T15%ash	Mix with 15% waste and higher ash content (CCA)
Phase VI	Phase III Mixes	TReference3, T5%, T10% and T15%
	Mixes from Phase V	REFERENCE5, T15% ash and T15%

The study commenced with the TReference Mix, labeled as TREFERENCE1, TREFERENCE2, etc., replicated in each Phase to minimize climatic variations. The mixing sequence initially combined gravel, sand, and half of the calculated water to enhance water absorption by the aggregates. Residues were added for mixes, incorporating them at this stage. Cement, micro-silica (or rice husk ashes), and the remaining water were added, followed by the gradual inclusion of the superplasticizer additive. The material achieved the desired workability after appropriate mixing and diluting the additive. This blend was then molded into cylindrical (11 cm × 21 cm) and prismatic (16 cm × 16 cm × 52 cm) samples for testing, and subjected to curing and surface treatments for analysis. In Phase I, designated "Determination of the Reference Mix," the CAD test mix aimed to examine additive behavior against aggregates. Four mixtures—T1, T2, T3, and T4—were created to achieve strengths exceeding 40 MPa. T1, T2, and T3 were adopted, while T4 was experimental. T1, the standard construction mix, was chosen, and T2 and T3 varied in sand and gravel content. Fresh state consistency was measured, and specimens were cured for analysis, focusing on axial compressive strength. Phase II aimed to determine rubber waste granulometry for replacement in the chosen Mix from Phase I. Rubber waste was separated into three sizes to substitute 5% of sand volume. The granulometries—Mix Tb less than 0.6 mm, Stroke Tb equal to 0.6 mm, Mix Tb greater than 0.6 mm—were examined for consistency and axial compressive strength. In Phase III, investigating the impact of residue percentage on axial compressive strength, variations of 5%, 10%, and 15% were tested against the reference mix. Fresh state consistency, curing, and tests for axial compressive strength were conducted. Phase IV compared Phase I and III Mix concerning curing and surface treatments' influence on axial compressive strength. Curing involved shadow and sunlight treatments, while surface treatments included cementation, plywood boards, surface capping, grinding, and rubber plates. Phase V assessed treatments to counteract residue-induced strength reduction, comparing traces from Phase I and III and introducing increased rice husk ash content in one of the mixes. Finally, Phase VI focused on analyzing damping coefficients by examining concrete with rubber in the CAD and with increased fine content from mineral additives in the CAD. Tests were conducted using Phase III and V Mix to evaluate the vibration's impact.

## Results and discussion

Using different types of crushed tire rubber instead of natural sand had clear effects on the properties of the concrete. The concrete's workability, hardness over time, and response to movement were all considered. When the rubber was mixed into the concrete, there was a slight decrease in its ability to flow, known as "slump." This was due to the shape and surface of the rubber pieces. Despite this, the concrete performed well for regular building and compacting. The weight of the concrete also decreased by about 2% when using 15% rubber because rubber pieces are lighter than natural sand. After the concrete had time to harden, its strength and flexibility were tested. Compared to regular concrete, the strength decreased by 14%, 28%, and 51% when replacing 5%, 10%, and 15% of the sand with rubber. However, all mixtures retained enough strength for many uses. The flexibility of the concrete also decreased, especially with 15% rubber. The concrete's response to the movement was examined. With 15% rubber, it was a bit less rigid but better at resisting vibrations. Examining the concrete's structure, changes were observed around the rubber particles. Using crushed tire rubber in concrete instead of sand can improve its ability to absorb vibrations while maintaining sufficient strength, especially with less than 10% rubber. Choosing smaller rubber pieces helps preserve workability and strength. With the right mix and treatment, this method could be valuable for recycling old tires in constructing robust structures.

### Phase I—determination of the reference mix

The study began with an experimental mix (1:2:3—cement:sand: gravel, by mass) using a satisfactory Superplasticizer proportion, increased by 0.5% of the cement volume. Additionally, microsilica was included at 8% of the cement mass. This resulted in a desired slump of 7.0 ± 2.0 cm for concrete with a water/cement ratio 0.37. Subsequently, various water/cement ratios and different cement, sand, and gravel ratios were explored for each mix to maintain the desired 7.0 cm ± 2.0 slump. A water/cement ratio of 0.3 was adopted, with water added incrementally (0.02 per cement basis each time). The cone trunk test was conducted after each water addition, and specimens were taken when the desired slump value was reached. Table 3 presents the mixes based on mass and their respective slump values.

**Table 3:** Mix originated in Phase I.

Traces	Cement	Sand	Gravel 0	Silica fume	Plasticizer	Water	Abatement (cm)
T1 Mix	1.00	2.00	3.00	0.08	5.48 × 10^−3^	0.38	5.0
T2 Mix	1.00	3.00	3.00	0.08	5.48 × 10^−3^	0.50	5.0
T3 Mix	1.00	2.00	2.00	0.08	5.48 × 10^−3^	0.39	8.5
T4 Mix	1.00	1.15	3.15	0.08	5.48 × 10^−3^	0.32	8.0

When dealing with larger quantities of material, especially with more sand, a higher water-to-cement (w/c) ratio was necessary to achieve the same slumps. This observation is evident in the T2 Mix compared to T1. Similarly, with a similar aggregate-to-cement (a/c) ratio, the T3 Mix exhibited greater workability than T1 for the same reason. Conversely, for the T4 mix, the smaller sand (fine aggregate with a greater surface area) required less water for increased fluidity. The resistance data, measured in MPa, are presented in Table 4.

**Table 4:** Data obtained in Phase I.

Cylindrical specimens 11 cm × 21 cm	fc	(MPa)	f_ct.sp_ (MPa)
	7 days	98 days	98 days
T1	37.33	62.1	4.54
	33.93	65.83	3.2
	34.46	52.16	3.74
Average ± deviation	35.24 ± 1.83	60.03 ± 7.07	3.82 ± 0.68
Coefficient of variation	5.19%	11.78%	17.80%
T2	27.96	40.6	4.22
	28.94	45.44	2.45
	29.48	30.36	2.95
Average ± deviation	28.79 ± 0.77	38.80 ± 7.7	3.20 ± 0.91
Coefficient of variation	2.67%	19.84%	28.43%
T3	40.22	36.04	3.89
	39.83	41.21	2.95
	40.64	47.38	3.41
Average ± deviation	40.23 ± 0.41	41.54 ± 5.68	3.42 ± 0.47
Coefficient of variation	1.02%	13.67%	13.74%
T4	40.55	50.19	5.51
	45.43	51.02	4.57
	51.98	49.35	5.43
Average ± deviation	$45.99 ± $5.74	50.19 ± 0.84	5.17 ± 0.52
Coefficient of variation	12.48%	1.67%	10.05%

**Figure 7:**
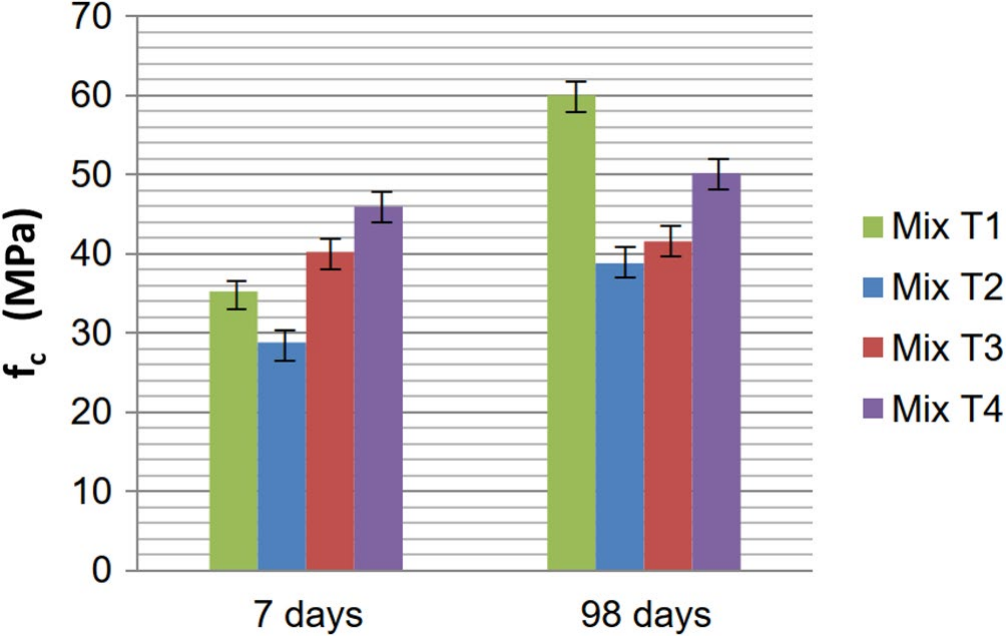
Behavior of the axial compressive strengths of the Phase I Mix at 7 and 98 days.

The averages of the axial compressive strength test values are illustrated in Fig. 7, at two different ages (in MPa).

**Figure 8:**
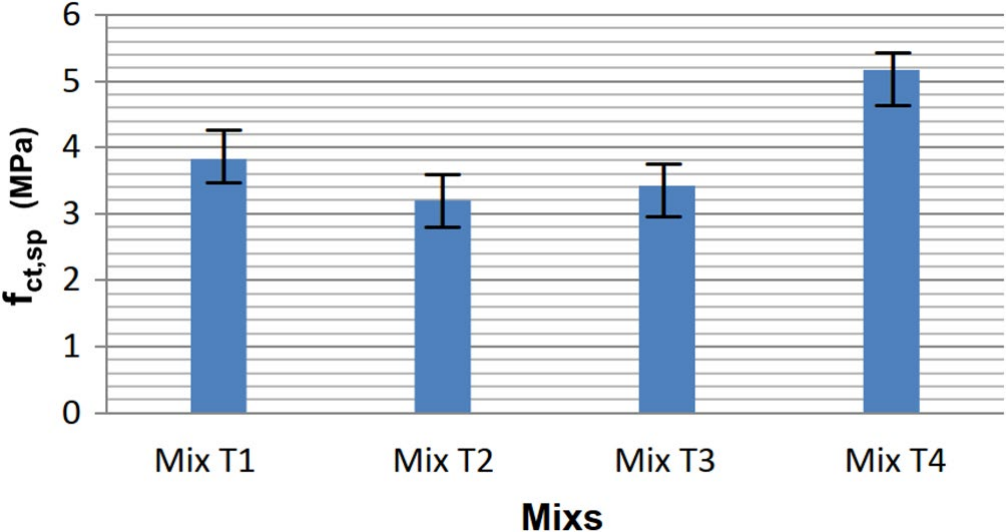
Diametrical compressive strengths of Phase I, at 98 days.

A notable increase in axial compressive strength was observed across the mixes from 7 to 98 days. The T2 Mix, with the lowest cement content and the highest water/cement ratio, displayed the weakest strength due to these factors. Mix T3, being richer in cement but with the second-highest water/cement ratio, showed strength close to Mix T2. Tensile strength through diametrical compression was measured only at 98 days, and the averages are illustrated in Fig. 8.

The T4 Mix demonstrated the highest tensile strength by diametrical compression at 5.17 MPa, surpassing T1 (3.82 MPa), which held the second position. Following, in descending order, were the T3 and T2 Mixes. Mean masses of the specimens in grams, along with their standard deviations, are presented in Table 5.

**Table 5:** Means, increase, deviation, and percentage of mass increase of the specimens.

Data from specimen batches	Mix T1	Mix T2	Mix T3	Mix T4
Average mass (g)	3802.1	3608.3	3642.1	3852.2
Standard deviation from the mean (g)	51.2	32.0	40.7	67.0

The data reveals that Mixes with higher sample masses (T4 and T1) exhibited superior resistance to axial compression and tensile strength by diametrical compression. This correlation is directly linked to their larger resistant section achieved by having smaller voids. The decreasing order of specimen masses aligns with the decreasing order of resistance for axial and diametrical measures. The better resistance observed in the T4 Mix at 7 days can be attributed to its lower water/cement ratio (compared to other mixes), higher cement consumption, and greater gravel ratio. Mix T3 also demonstrated high resistance at 7 days, which can be attributed to various factors, including the water/cement ratio and consumption. In pursuing a mix with resistance exceeding 40 MPa at 7 days, both T3 and T4 met this criterion. Considering T4 achieved higher strength with lower cement consumption, it was chosen as the study's reference Mix ("Base Mix"). Unfortunately, data at 28 days was unattainable due to testing machine issues, so the 7-day data was considered.

### Phase II—determination of the particle size of the residue to be used

Redone the T4 Mix, which will be called TReference2, The Replacement of sand by 5% of waste, by volume, for the different granulometries of the rubber. The proportions (in mass) of the materials relative to the Mixes are shown in Table 6.

**Table 6:** Phase II mixes with residues.

Cement	Sand	Rubber	Brita zero	Silica fume	Superplasticizer	Water
1.00	1.09	0.02	3.15	0.08	5.48 × 10^−3^	0.32

In this Phase, the cement consumption was around 464 kg/m^3^, consistent across the rubber mixes and TReference2. The slump values, measured using the cone trunk, were similar for all four mixes, ranging from 4.0 ± 2.0 cm. Table 9 displays the averages of specimen masses in grams and their respective standard deviations. The results for axial compressive strength at 56 and 224 days and tensile strength by diametrical compression at 56 days are presented in Table 7.

**Table 7:** Values obtained in Phase II.

Cylindrical CPs 11 cm × 21 cm	fc	(MPa)	f_ct.sp_ (MPa)
	56 days	224 days	56 days
REFERENCE2	37	52.11	3.86
	35.76	59.22	4.58
	35.42	57.63	4.14
Average ± deviation	36.06 ± 0.83	56.32 ± 3.73	4.19 ± 0.36
Coefficient of variation	2.37%	7.00%	8.59%
Tb < 0.6 mm	30.61	54.34	4.14
	28.77	52.64	4.21
	26.68	53.57	5.02
Average ± deviation	28.69 ± 1.97	53.52 ± 0.85	4.46 ± 0.49
Coefficient of variation	6.87%	1.59%	10.99%
Tb = 0.6 mm	39.33	60.11	3.86
	28.76	54.79	4.66
	32.39	56.41	5.25
Average ± deviation	33.49 ± 5.37	57.10 ± 2.73	4.59 ± 0.70
Coefficient of variation	16.03%	4.78%	15.25%
Tb > 0.6 mm	38.42	48.24	4.04
	36.57	56.05	4.92

**Figure 9:**
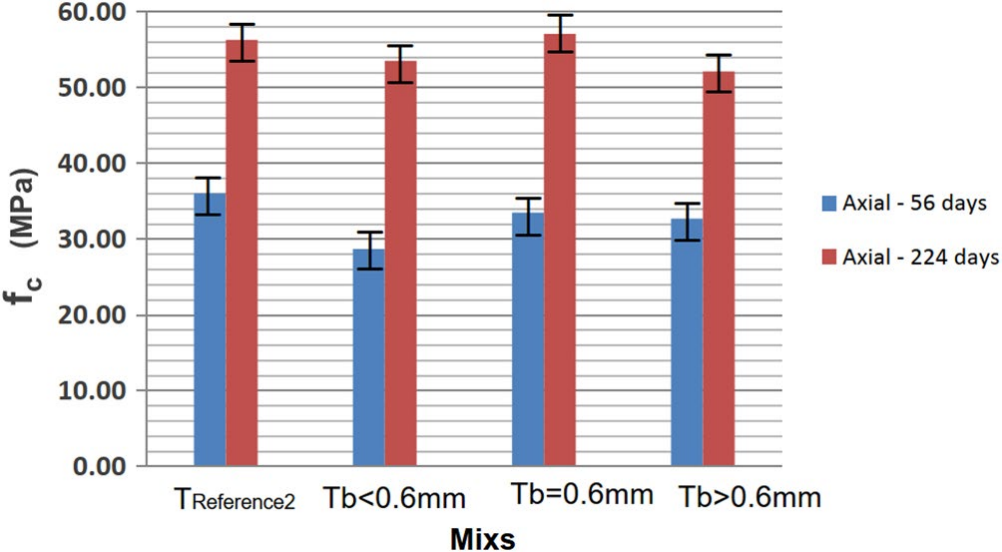
Mean axial compressive strengths at 56 and 224 days.

Including rubber in the concrete resulted in a slight reduction in axial compressive strength compared to the reference concrete. However, over time, the rubber in the mixture demonstrated a more significant increase in resistance, even surpassing the reference in the case of Mix Tb = 0.6 mm (57.10 MPa). This trend is better illustrated in Fig. 9.

**Figure 10:**
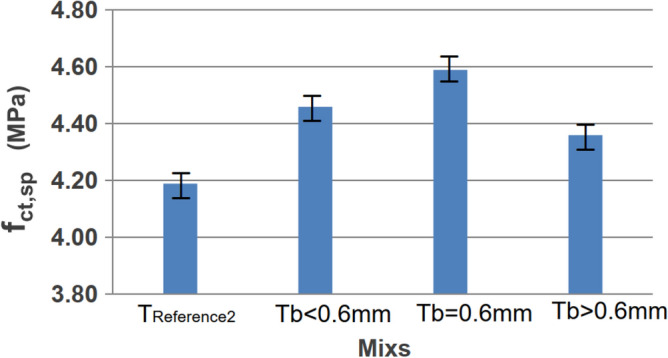
Diametrical resistances at 56 days.

The highest standard deviation was observed for the mix with the highest rubber residue for both ages. This can be attributed to the lower uniformity of the residues, leading to varied properties among the produced specimens. The differences in strengths can be explained by varying deformations of the grains when subjected to loading or different accommodations in the voids between the component grains of the concrete. In the case of tensile strength by diametrical compression, there was a slight increase (or approximate continuity of the value) with the addition of residues, suggesting that, for this aspect, the material had less influence. Figure 10 illustrates the behavior of tensile strengths by diametrical compression, comparing different proportions of rubber residue at 56 days.

### Phase III—determination of the percentage of the residue in the mix to be treated

**Figure 11:**
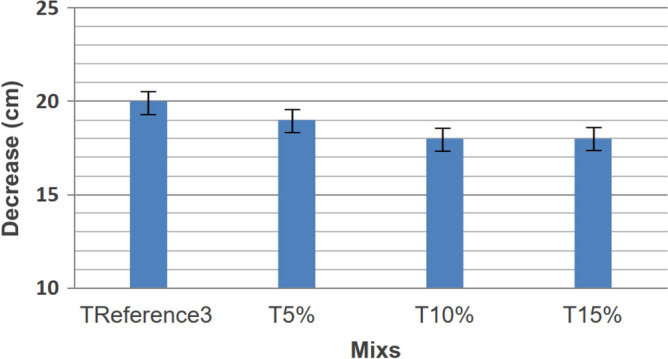
Slump test.

In this third Phase, rice husks were used as a replacement for silica fumes due to the insufficient availability of silica fumes to complete the work. This substitution did not result in noticeable or significant changes, and the results of this Phase are not compared with those of Phase II. Only rubber with a particle size equivalent to 0.6 mm (obtained in phase II) was used in this Phase, as it demonstrated better results in axial compressive strength and tensile strength by diametrical compression compared to other granulometries. There was a significant reduction in fluidity as the amount of residues in the composition increased, depicted by a consistent decrease in slump, as shown in Fig. 11.

The reduction in slump may be attributed to the surface friction between the material and the rubber particles, aligning with the findings of Reda Taha et al. (2008). Table 8 presents the axial compressive and tensile strength values in diametrical compression at 7 and 120 days, measured in MPa.

**Table 8:** Phase III resistance values.

Cylindrical CPs 10 cm × 20 cm	fc (mpa)		f_ct.sp_(mpa)	
	7 days	120 days	7 days	120 days
T15%	28.03	42.75	1.97	2.50
	30.11	39.39	2.04	3.35
	29.53	48.10	3.03	3.20
Average ± deviation	29.22 ± 1.08	43.41 ± 4.39	2.35 ± 0.59	3.01 ± 0.45
Coefficient of variation	3.69%	10.11%	25.10%	14.95%
T10%	40.43	49.03	3.77	3.44
	39.50	49.79	3.51	4.09
	39.92	49.30	2.37	3.91
Average ± deviation	39.95 ± 0.46	49.37 ± 0.39	3.22 ± 0.75	3.81 ± 0.34
Coefficient of variation	1.15%	0.79%	23.29%	8.92%
T5%	46.75	49.37	2.79	4.10
	41.66	42.39	3.12	4.30
	38.48	59.44	2.48	3.86
Average ± deviation	42.30 ± 4.17	50.40 ± 8.57	2.79 ± 0.32	4.09 ± 0.22
Coefficient of variation	9.85%	17.00%	11.47%	5.38%
REFERENCE3	45.24	64.24	2.76	4.78
	38.71	58.73	2.91	4.89
	38.64	52.96	2.95	4.12
Average ± deviation	40.86 ± 3.79	58.64 ± 5.64	2.87 ± 0.10	4.6 ± 0.42
Coefficient of variation	9.28%	9.62%	2.17%	9.13%

**Figure 12:**
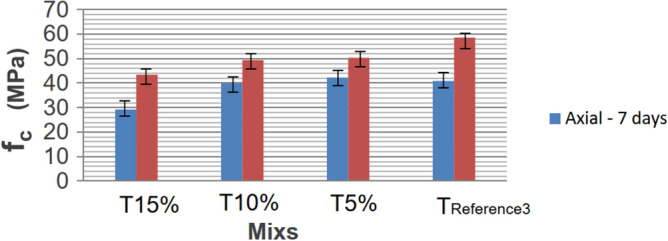
Axial compressive strengths at 7 and 120 days, for Mixscom and without rubber.

**Figure 13:**
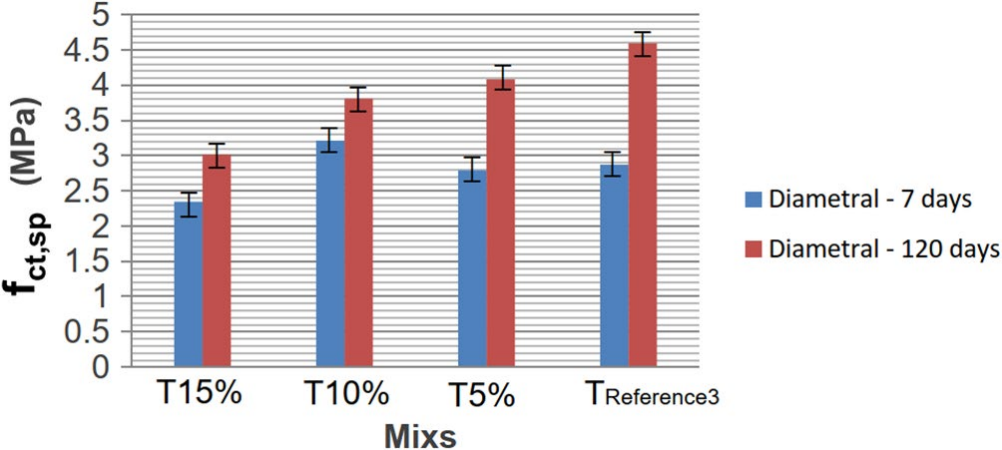
Tensile strengths by diametrical compression at 7 and 120 days, for the mixes with and without rubber.

In broad terms, the resistances (axial and diametrical) exhibit similar influences with the addition of rubber while maintaining the proportions. Figures 12 and 13 illustrate the generated curves, depicting the increase in the substitution of sand with rubber waste, progressing from TREFERENCE3 to T15%.

The data averages at seven days indicate that an increase in rubber residues up to 10% had minimal influence. The strength of 5% rubber (T5%) was practically the same, with the concrete having 1.44 MPa more than TREFERENCE3. However, a noticeable decrease became evident for 15% rubber, showing 11.64 MPa lower strength than the reference mix. After 120 days, axial compressive strengths exhibited greater discrepancies among the mixes. However, the mix with rubber showed a higher standard deviation, potentially reaching the mean value of the mix without rubber. The overall trend indicates a decrease in strength with increased rubber residues. This reduction in resistance can be attributed to rubber's lower compressive strength, where the residues function similarly to voids in the concrete or create tensions due to their elliptical shape when compressed.

For tensile strength by diametrical compression, the drop in resistance may result from reduced friction or the lack of adhesion between rubber particles and concrete, supporting previous research. When comparing rupture ages, the addition of 15% rubber in the concrete (T15%) showed a 28.5% reduction in axial compressive strength at seven days compared to concrete without rubber (TREFERENCE3). At 120 days, the reduction was 26%, indicating a consistent difference between the reference concrete and the concrete with 15% rubber at both ages.

**Figure 14:**
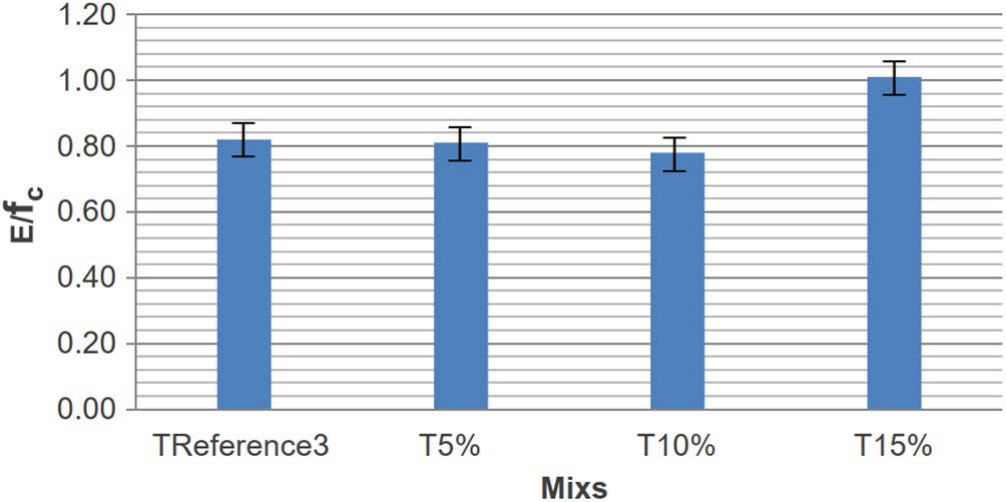
Modulus of elasticity/axial compressive strength T5%, T10%, T15% and T_REFERENCE3_.

These findings were based on the choice of the mix with 15% rubber residues for treatment in phase V. The modulus of elasticity, obtained one hundred and sixty-nine days after sample production, also supported the evaluation of material ductility. These values confirmed that axial compressive strengths had little influence when the residue percentage reached 10%, even more than 24 weeks after manufacture. The standard deviation was high in the mix with 5% substitution (T5%), resulting in a lower axial mean than T10%. The "Modulus of Elasticity/Axial Compressive Strength" ratio is depicted in Fig. 14.

The addition of rubber tends to decrease the modulus of elasticity of concrete, resulting in increased deformation under the same load for rubberized concrete. In this context, the T10% Mix exhibited the most favorable relationship for gaining ductility while minimizing resistance loss, as it had the lowest modulus of elasticity/axial compressive strength ratio. The mix with 5% rubber also showed a lower ratio than the reference mix. The natural sand has a consistent gradation ranging from 0.15 to 4.8 mm, resulting in a fineness modulus of 2.66. On the other hand, the fine rubber fraction passing the 0.6 mm sieve concentrates within the 0.3 mm to 0.6 mm range, while the medium rubber fraction (0.6–1.19 mm) centers on 0.6 mm to 1.0 mm particles. Coarse rubber particles (1.19–2.36 mm) span approximately 1.0 mm to 2.0 mm. Although the three rubber fractions cover the overall size distribution of the original sand, they are divided into narrower bands. Notably, the fine rubber has a limited maximum size of around 0.6 mm, unlike the sand's nearly 5 mm. There is a noticeable gap in particle sizes between the coarse and medium rubber fractions, approximately around 1.0–1.2 mm. Essentially, the crumb rubber particle size distributions align with sections of the sand gradation curve but are separated into finer size ranges during sieving.

### Phase IV—analysis of certain material properties

**Figure 15:**
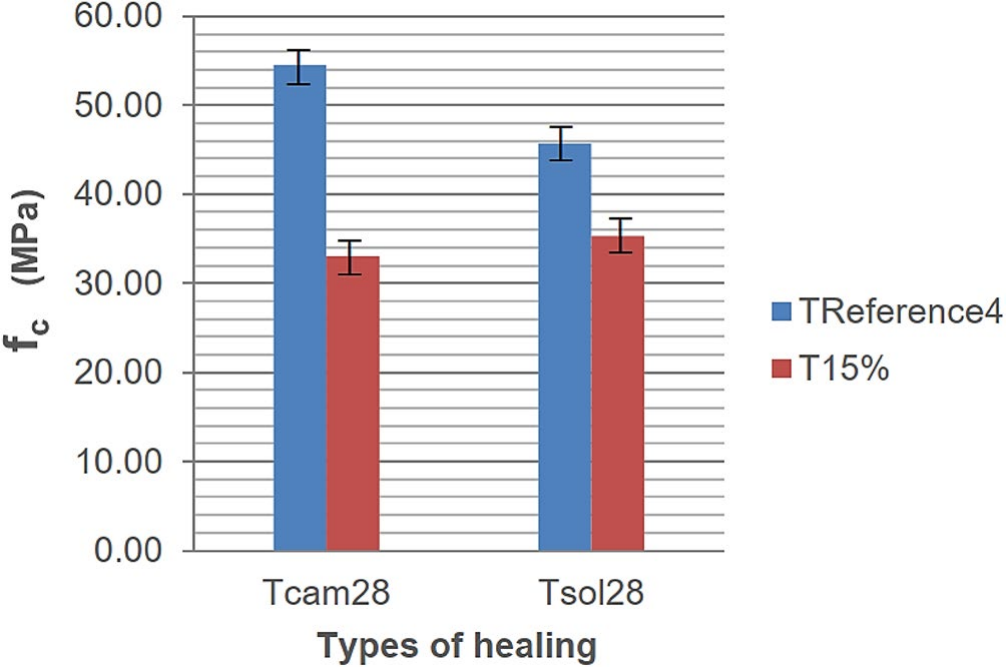
Axial compressive strengths obtained.

The remade mixes in this section included TREFERENCE1 from Phase I, now referred to as TREFERENCE4 and the T15% mix from Phase III. It is observed that the curing treatment (process) had a minimal influence on the CAD mix with rubber. In contrast, for the non-rubber CAD mix, different curing types resulted in differences of nearly 8 MPa. However, unlike the rubber mix, the resistance decreased when curing under the sun. Figure 15 illustrates the average resistance behavior of the batches in the two different curing processes for 28 days.

Concrete with residues exhibited similar behavior (only 6.9% higher) under the sun. In contrast, reference concrete showed lower strength values (16.1% lower) when cured under the sun, indicating the influence of curing methods on mechanical properties. The reference concrete, suffering greater shrinkage under the sun, suggests that rubber concrete, being more ductile, accommodates better to shrinkage. In contrast, rigid concrete tends to crack during retraction. Surface treatment analysis on axial compressive strength was conducted with 15% rubber residue mix.

The top three treatments were Cemented, Plywood, and Capped. Rectification treatment showed significant discrepancies, likely due to multiple planes formed on the specimen surface, reducing load application area and increasing tension. Proper calibration of the grinding machine is crucial. Using rubber plates for specimen rupture resulted in lower mean values (below 14 MPa) with a more consistent standard deviation than grinding. The Poisson effect and rubber flow perpendicular to the load plane, combined with adhesion, may increase cracking with load increase. Cementation treatment yields favorable results with similar mechanical properties and good adhesion. Plywood deforms with load, providing optimal load transfer during testing. Sulfur capping, though reasonably accommodating like cementation, depends on sulfur quality. Sulfur, being a different material, may influence stress passage to the sample, explaining the drop in strength compared to cementation. The timing of sulfur treatment affects its hardness, impacting strength.

### Phase V—concrete treatments with 15% waste

Analyses for the mixes (t15%, treference5, t15% ash) were conducted in both the fresh and hardened phases. The results were presented in the following two sub-items. Mass measurements of the samples were taken to correlate with mechanical behaviors. Table 9 displays the averages and standard deviations for cylindrical and prismatic samples.

**Table 9:** Average masses of the specimens produced.

Type of CP	CP batch data	REFERENCE5	T15%	T15%Ash
Cylindrical	Average mass (g)	3780.00	3730.00	3710.00
	Standard deviation from the mean (g)	0.05	0.05	0.04
Binoculars	Average mass (g)	28,810.00	28,200.00	27,020.00
	Standard deviation from the mean (g)	0.22	0.03	0.33

**Figure 16:**
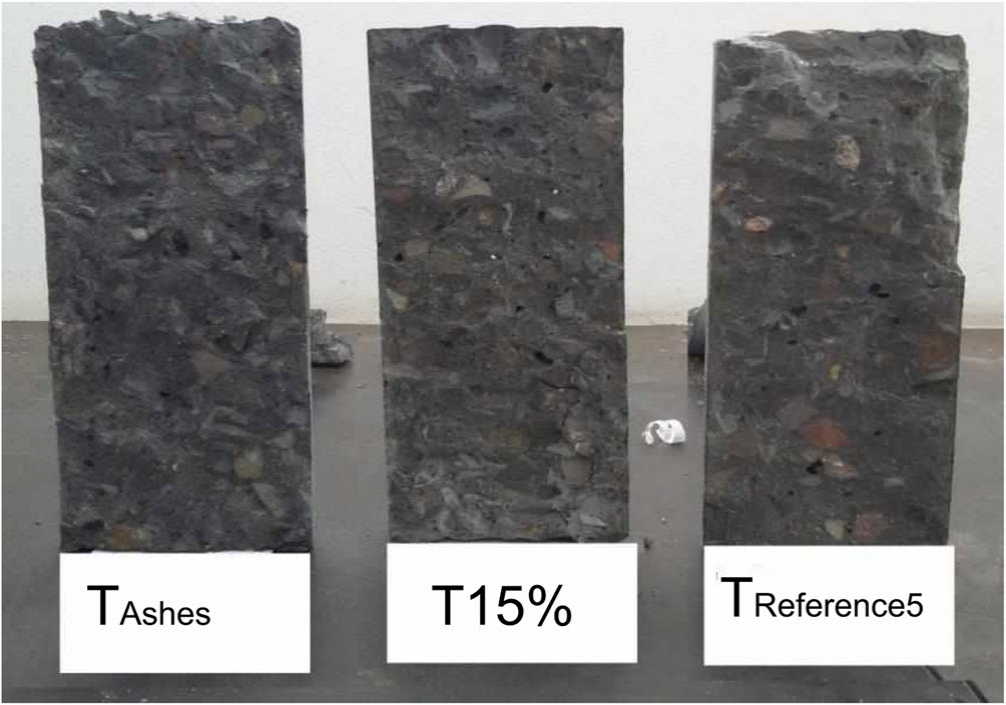
Three samples, one from each mix, with internal detail.

Waste insertion resulted in a lighter material. As expected, TREFERENCIA5 had the highest mass with no residues, followed by T15%. Surprisingly, T15% Ash, despite having finer ash (rice husk ash), exhibited the lowest mass. For cylindrical samples with lower standard deviation, waste insertion caused a 1.32% mass decrease, while combining waste and ash led to a 1.85% decrease. In this study, mix with a rubber rate of 12.0 kg/m^3^, self-weight reduction in the structure was observed due to the specific masses of materials, specifically sand and rubber. Figure 16 depicts longitudinally broken samples from each mix after the tensile strength test by diametrical compression, illustrating minimal vibration and no segregation in the fresh Phase for all three mixes.

**Figure 17:**
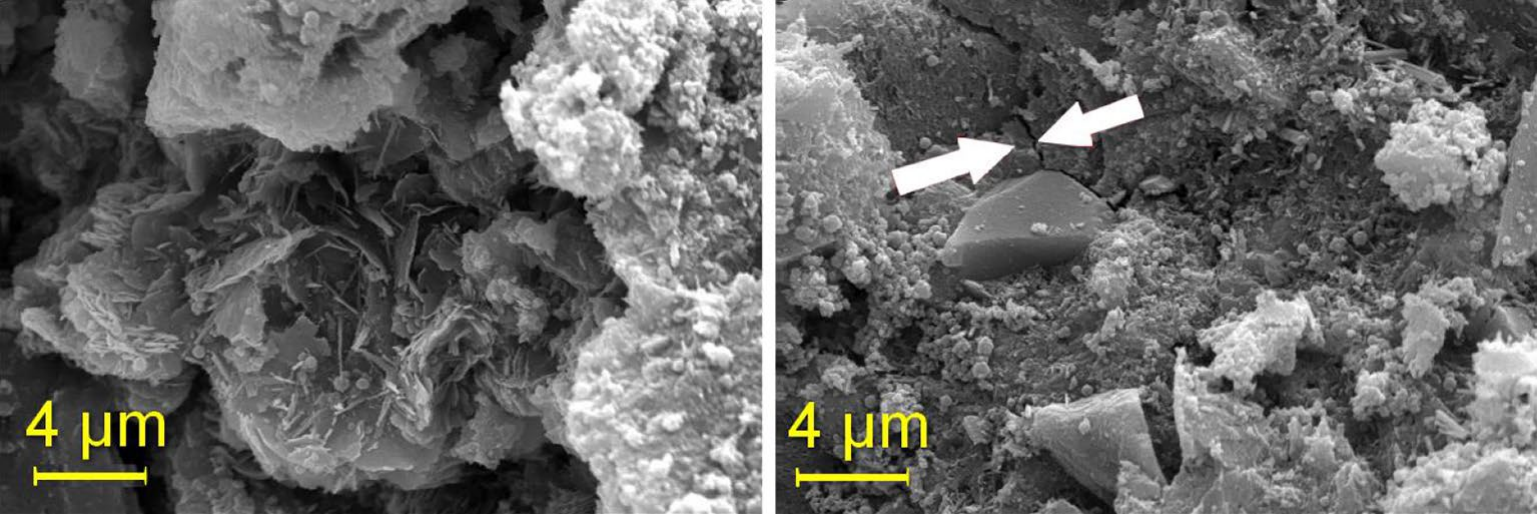
T15% Mix microscopy.

**Figure 18:**
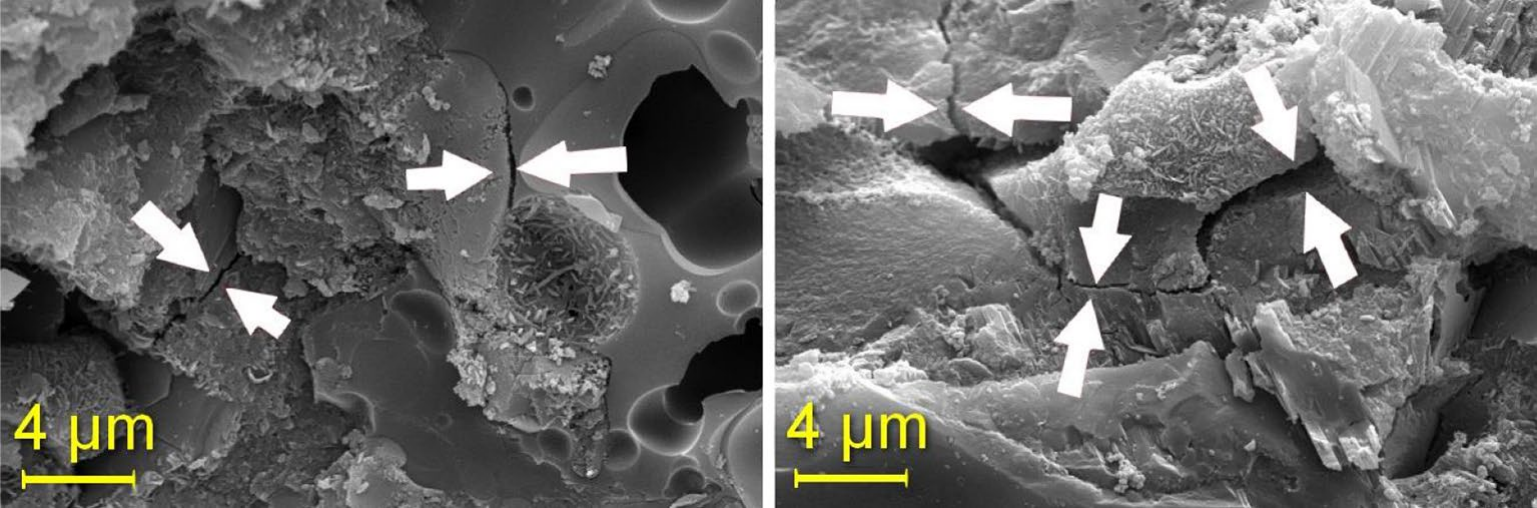
Microscopy of the T15% ash mix.

**Figure 19:**
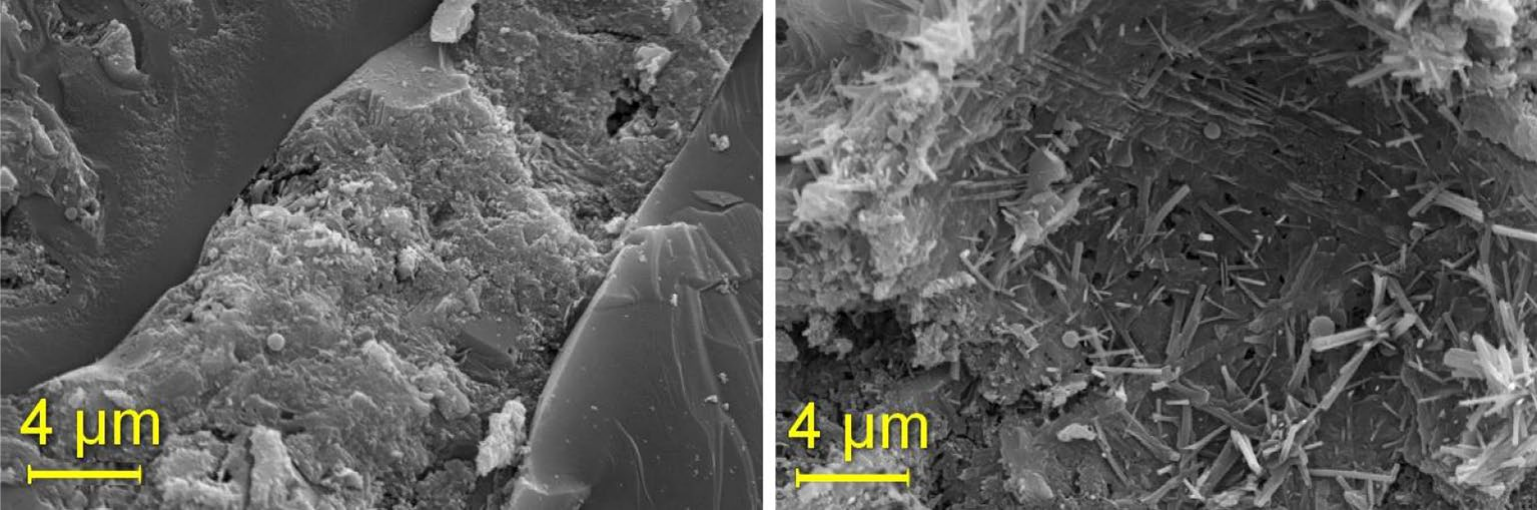
Mix reference 4 microscopy.

In concrete pastes with 15% rubber, micro-cracks become apparent at 28 days. Conversely, rubber-free concrete exhibits better "accommodation" between the paste and aggregate particles. The images captured represent two distinct regions (images a and b). Figure 17 illustrates the T15% Mix, while Fig. 18 showcases T15% Ashes. TReference5 is presented in Fig. 19.

In concrete with higher ash content, cracks, including micro-cracks formation, are more pronounced, as depicted on the right in Fig. 18. The Figure highlights the significantly smaller cracks observed in concrete with an ash content of 8%. Moving to Fig. 19, the absence of noticeable ettringite formation and fissures is evident in the photographed regions. However, the image emphasizes the excellent particle adhesion in the mix without rubber.

## Conclusions

This study investigated the effects of substituting waste tire rubber particles for natural sand on high-performance concrete (HPC) properties. Based on the experimental results, the following conclusions can be drawn:

- Replacing sand with up to 10% rubber by volume caused a minimal reduction in compressive strength, tensile strength, and modulus of elasticity compared to plain HPC. With 15% replacement, the strength and modulus losses were more substantial at around 30–50%.
- The addition of rubber increased the ductility and toughness of HPC, evidenced by the greater energy absorption before failure. The damping ratio increased markedly with rubber content, which benefits vibration resistance.
- Smaller rubber particles (passing 0.6 mm sieve) were more effective than larger sizes in mitigating adverse impacts on strength and workability. Finer rubber particles can fill voids similarly to sand.
- Increasing the fine content by adding more silica fume did not prevent the strength reductions with 15% rubber replacement. The higher fines likely disrupted particle packing.
- Surface treatments like sulfur capping were valuable for minimizing end effects and improving load distribution during compressive strength testing.
- Overall, controlled replacement of sand with waste tire rubber aggregate appears feasible in HPC up to 10% by volume substitution. This can enhance characteristics like damping and toughness while retaining adequate strength.

Further research can explore combining rubber particles with fiber reinforcement or oxidizing treatments to maintain strength. Testing the long-term durability and shrinkage behavior will be important. Investigating the dynamic properties and vibration mitigation more extensively could support uses in impact and earthquake structures. With additional study, waste tire rubber has good potential to improve sustainability in the growing application of HPC for construction worldwide.

### Supplementary Information

Supplementary Information.

## Data Availability

All data generated or analysed during this study are included in this published article [and its supplementary information files].

## Abbreviations

HPC: High-performance concrete
w/c: Water-cement ratio
CP: Cylindrical specimen
fc: Compressive strength
fct,sp: Splitting tensile strength
E: Modulus of elasticity
