# Supplementary material for: Enhancing high-performance concrete sustainability: integration of waste tire rubber for innovation
Source: Sci Rep. 2024 Feb 26;14:4635. doi: 10.1038/s41598-024-55485-9 (PMC10897324; doi:10.1038/s41598-024-55485-9)
Supplement: Supplementary file 1 — Supplementary Information. [file 41598_2024_55485_MOESM1_ESM.docx]

**Materials characterization tests**

The determination of its apparent specific mass was determined according to ABNT NBR 52:2009. In addition to the oven for sand drying, this test used the following equipment: precision scale, conical trunk mold, socket, pycnometer, tray, spatula, funnel, and brush. The sample was placed in a container where it remained covered by water for 24 hours (resting). Subsequently, the excess water was removed, and an air current was allowed to pass through so that the sample's humidity could be regulated. The procedure was repeated to verify it: the wet sand was placed on the conical trunk, and its surface was compacted with 25 blows applied with the socket. When the landslide occurred, the sand was in the saturated condition of dry surface.

Thus, 500 grams of the sample was placed in the pycnometer, which was completed up to 500 cm³. The particle size for the fine aggregate was determined according to ABNT NBR NM 248:2003. For this test, the following were used: sand container, precision scale, vibrating table with sticks for fixing the sieves and sieves with different openings. The [Figure 1 a](#bookmark39)) and b) show the procedure: the material, with zero moisture (0%), was separated and submitted to sieving in the appropriate laboratory equipment.


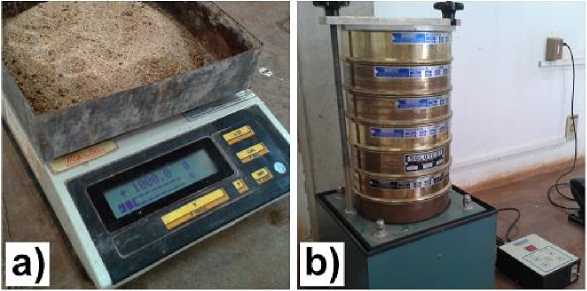


Figure 1 a) Separate material; b) Testing in progress

The specific mass was obtained according to ABNT NBR NM 53:2009. After obtaining the aggregate mass for testing with the precision balance, it was washed on a 4.8 mm sieve, placed in trays, and dried in an oven until the mass was constrained. It was waited for cooling until it was possible to handle the material (about 50 °C). Then, the material was immersed in water using a bucket, remaining for (24±4)h. The sample was removed from the water and spread on a cloth to dry its surface. Care was taken not to evaporate water from the pores, so the mass of the sample was measured in a saturated condition with a dry surface. After the surface drying, the sample was placed in a hollow container to be completely immersed in water without material loss. The container was attached to the scale plate, located at a higher level than the water bucket, where the sample was immersed. The set of scales plus the container was previously zeroed, empty, and immersed in water. Then, the submerged weight was obtained. The specific mass was calculated with the three measurements. Two of the steps in the process are represented in the [Figure 2 (a](#bookmark43)) and [Figure (b](#bookmark43)).


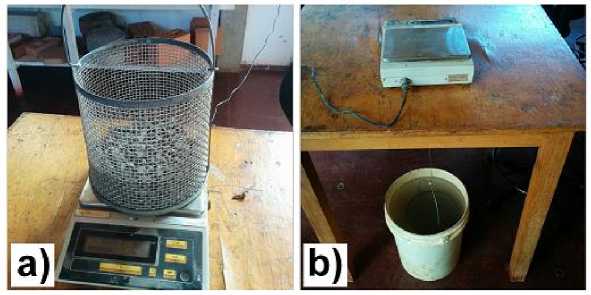


Figure 2 (a)Separated gravel; b) Testing in progress

We proceeded according to ABNT NBR NM 248:2003 to determine the particle size size. A large mechanical agitator with sieves was used. A 6.5kg sample of the material was placed and vibrated for ten minutes. [Figure 3](#bookmark46) (a) shows the agitator used, and (b) shows the detail of a portion of the material retained in one of the sieves.


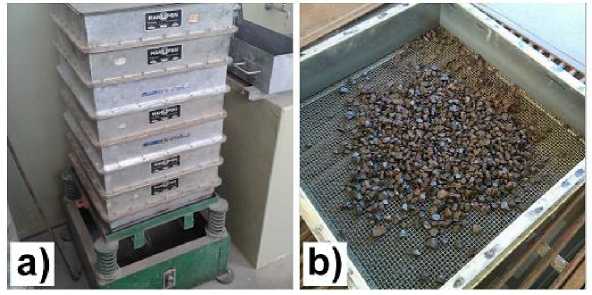


Figure 3 a) Aggregate agitator; b) Material in one of the sieves

No process was carried out to remove the gravel dust in the material, which by mass represents approximately 1% (obtained from a test with a certain sample) composed of particles passing through the 0.3 mm sieve.

The Chapman tube was used to determine its specific mass, analogous to that described in ABNT NBR 9776:1987. For this test, the following equipment was used: funnel, water container, Chapman tube, precision scale, sand container. A total of 50 g of rubber was placed in a Chapman tube with 380 cm³ of alcohol (in this case, no water was used due to the low specific mass of the rubber). At the end, the formula was read and changed. In order to separate only the granulometry desired for use, it was proceeded in a manner analogous to that of the gravel granulometry, using sieves corresponding to the desired ranges, between 0.3 μm and 2.36 mm. In this case, the analysis was carried out through the cone trunk slump test, following the ABNT NBR NM 67:1998 standard. The concrete was placed in a cone trunk in three layers, and in each of them, 25 strokes were given. (Cases 1, 2, and 3 of the [Figure 16)](#bookmark52). The trunk was shredded (4), lifted (5), and mass slump was measured (6). Measurements of the specimens' mass were performed to relate the voids to the properties in the hardened state.

**Hardened state**

The axial compressive strength value was obtained according to ABNT NBR 5739:2007. Cylindrical specimens were used for this purpose.  [Figure 4 a](#bookmark58)) shows the specimen being subjected to the test (start of the test) and (b) shows the broken specimen at the end of the test.


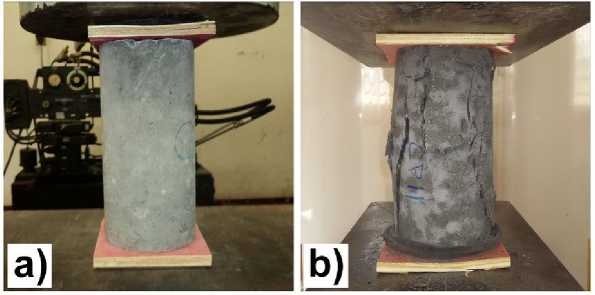


Figure 4 a) Beginning of uniaxial compression test; b) End of test

The tensile strength value was obtained with the diametrical compression tensile strength test, according to ABNT NBR 7222:2011. For this purpose, cylindrical specimens with a height equivalent to 20 cm and a diameter of 10 cm were used. Two wooden plates were placed diametrically at the upper and lower contact of the specimen with the steel mold. The [Figure 5(a](#bookmark62)) shows the assembly detail (specimen and steel mold) for testing. The [Figure 5 (b](#bookmark62)) shows a test specimen.


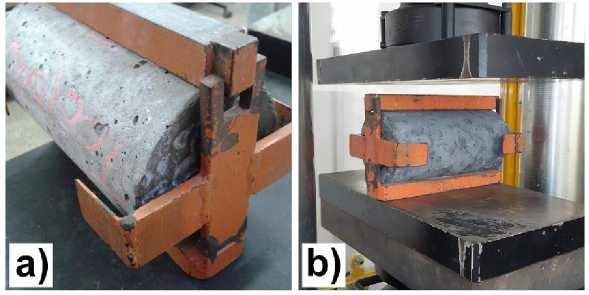


Figure 5 a) Set for diametrical compression tensile test; b)Trial starting

The value of the module was obtained according to the ABNTNBR 8522:2008 test.

Cylindrical specimens were used for this purpose. The [Figure](#bookmark64) 6 (a) shows a specimen prepared with the strain gauges, seen from above. The [Figure 6(b](#bookmark64)) shows the specimen being subjected to the test (already in progress).


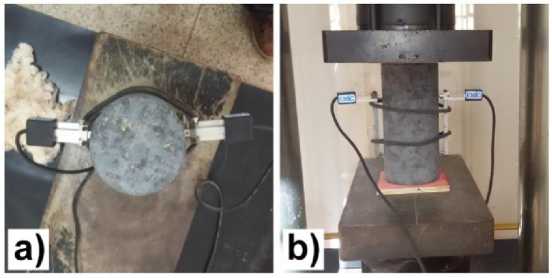


Figure 6 a) Extensometers in the specimen (top view); (b) Modulus of elasticity test

The tensile strength value in flexion was obtained by the test recommended by ABNT NBR 12142:2010, with a cleaver. For this purpose, prismatic specimens (measuring 15 cm x 15 cm x 50 cm) were used. The [Figure 7(a)](#bookmark68) shows a prismatic specimen at the beginning of the test. The [Figure 7(b)](#bookmark68) shows the break at the end of the test.


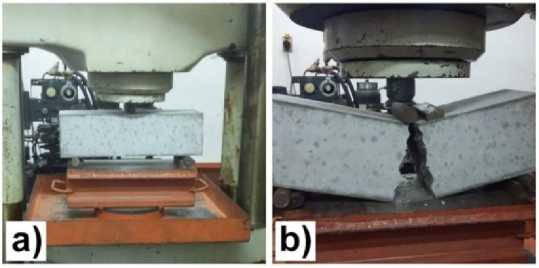


Figure 7 a) Beginning of flexure test; b) Ruptured specimen

The vibration test involves striking the impact hammer against the specimen, causing tension. This impact needs to have a minimum value in order to be readable. The waves are captured by the accelerometers (sensors) previously placed on the sample (utilizing a special glue, easily removable after the test). The [Figure 8(a)](#bookmark70) shows the hammer, while the [Figure](#bookmark70) (b) shows the accelerometers.


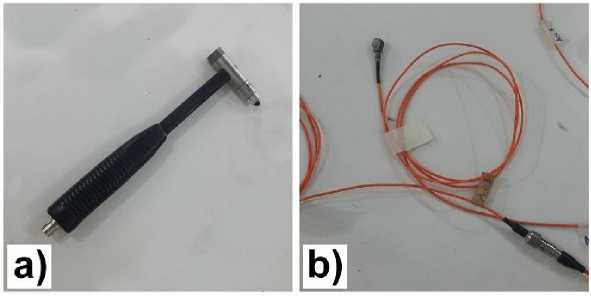


Figure 8 a) Impact hammer; b) Accelerometers

Due to the sensitivity of the receivers, an inertial table is used to avoid the influence of external frequencies on the test. The specimen (usually metal beams) is attached to it for the test to proceed. In the case of concrete, cylindrical specimens (10 cm x 20 cm) were used. It was decided to adapt the assay for the type of sample and material. Thus, the sample was set in the lathe nut, which had the same function as the inertial table.  [Figure 9](#bookmark71) shows the inertial table.


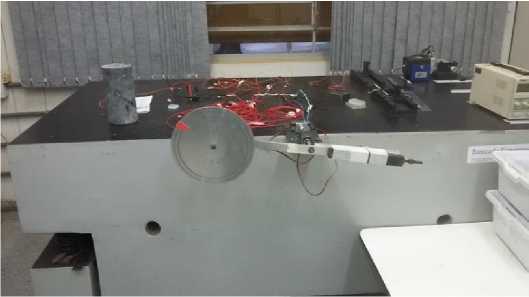


Figure 9 Inertial table

Figure 10 shows two views, (a) and (b), of the specimen attached to the lathe.


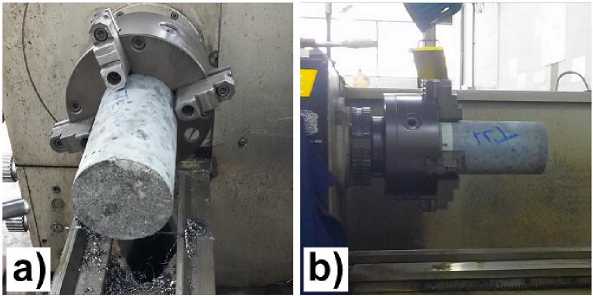


Figure 10 Specimen Crimped in the Lathe

After the specimen was set, the accelerometers were glued, allowing the excitation with the hammer to begin. Both the hammer and the accelerometers were linked to the acquisition system. The [Figure 11(a)](#bookmark73) shows the accelerometers glued to the sample. The acquisition system is shown in the 11(b).


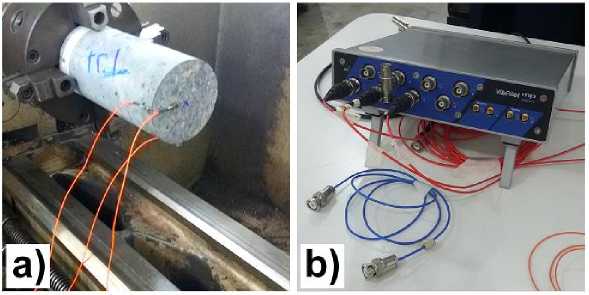


Figure 11 a) Sample prepared with the sensors; (b) Data acquisition system

In order to verify the homogeneity of the sample material (concrete), the behavior with blows was analyzed in different locations: on the upper face and in two distinct regions of the cylindrical side.Once the data were acquired, they could be processed using appropriate numerical analysis software (*Matlab*). Thus, obtaining the natural frequency and the values of the respective damping coefficients, in addition to the graphs, was possible. Testing is important to find structural responses through applied loads in a non-destructive manner. For the readings of the images to be performed in scanning electron microscopy, the samples needed a "gold bath", allowing the assay to be performed. Initially, the mixtures were separated on the supports so they could be taken to the metallizer (sputtering) and receive gold atoms. Care was taken to ensure a path for the electrons to exit the sample through a glued conductive tape. The conductive tapes on the brackets (approximately 1 cm in diameter) for the samples are shown in the [Figure](#bookmark77) 12(a) while the metallizer is shown in the [Figure 12(b)](#bookmark77).


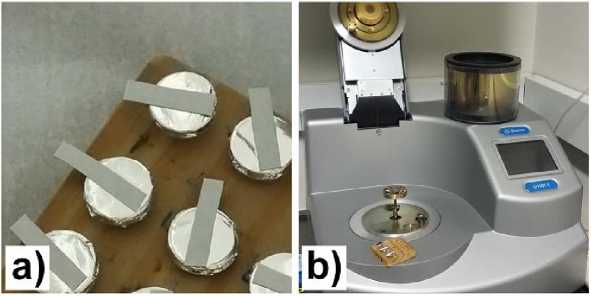


Figure 12 a) Samples prepared; b) Metallizer used

Once ready, the samples were placed under the microscope for further testing. In [Figure 26](#bookmark80) (a), the samples with the gold atoms on them, and in Figure 13 (a)the equipment (electron microscope) used.


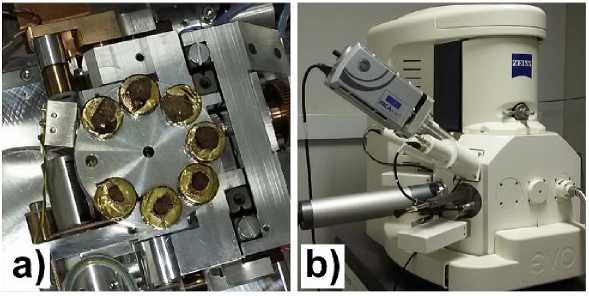


Figure 13 a) Post-treatment samples; b) Electron microscope

The images could be obtained from proprietary software on a computer attached to the microscope.

**Healing Methods**

During the work, two different types of cures were used. It was the standard type of cure in this research. It was present in all phases (which will be described in the experimental procedure). In this process, after demolding, the samples were placed on the shelf of the wet chamber of the FEIS/UNESP laboratory, where they remained until they reached the age relative to the tests. In [Figure 14a](#bookmark87)) is illustrated with the view of the damp chamber and in the [Figure 14](#bookmark87) b), the detail of the specimens on the shelves.


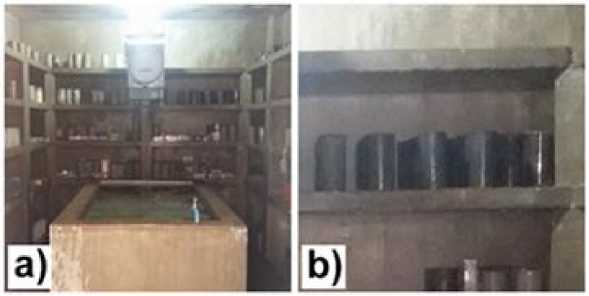


Figure 14 a) View of the wet chamber b) Specimens on the shelves

In the chamber, the temperature must be (23 ± 2)°C and the humidity must be at least 95% to ensure that the surfaces of the specimens are moist, according to ABNT NBR 9479:2006. However, in this study, the wet chamber stopped working after phase I curing and did not meet these conditions. In this procedure, after demolding, the samples were placed under the sun in the external courtyard of the FEIS/UNESP laboratory, where they remained until the age relative to the tests. In [Figure 15](#bookmark91) The procedure of curing under the sun is shown.


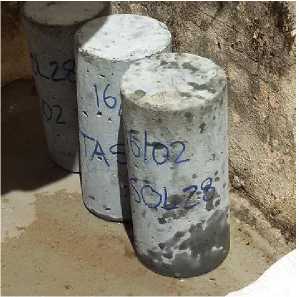


Figure 15 Specimens curing in the sun

**Surface treatments**

The flatness of the specimens is an important characteristic so that the distribution of axial loads in the tests is equal over the entire face in question. The surface treatment processes used in this research are described in this sub-item. They are compared with each other for the axial compressive strength values, since in the tensile tests in compression and flexion, it is unnecessary to treat the specimen. It was the most used option in this research. In this case, no treatment processes were necessary on the specimens, as it consisted of correctly coupling the 11 cm x 11 cm (0.9 cm thickness) plywood plates at the ends of the specimens when placed in the testing machine, at the time of the axial test. The plates are shown in the [Figure](#bookmark98) [16th](#bookmark98)), and the process in the [Figure 16b](#bookmark98)).


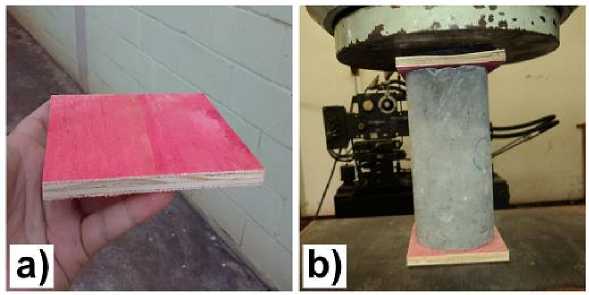


Figure 16 a) Plywood board; b) Specimen during plywood test

The original plate was 220 cm x 110 cm in size, and it was necessary to go through a cutting process through a 3 mm diameter saw. It was the second most used planning option in this research. Equipment from the FEIS-UNESP laboratory was used to carry it out. The sulfur was melted in the pot, located inside a vat with an exhaust fan called a "fume hood." The liquid sulfur is placed in a steel dish and the surface of the specimen is immersed in it, until solidification occurs (at room temperature) and, therefore, adhesion to the specimen.The instruments are shown in the [Figure 17](#bookmark103) a), The detail of the steel plate on the [Figure 17](#bookmark103) b) and the result in the [Figure 17](#bookmark103) c).


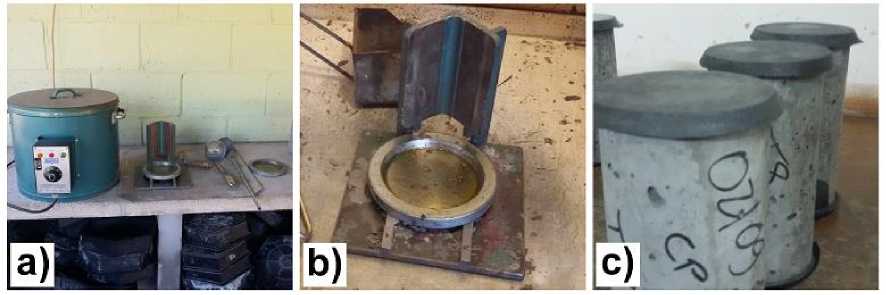


Figure 17 a) Instruments used in capping; b) Steel plate; (c) finalized specimens

It was made possible by employing the rectifier machine of the FEIS-UNESP laboratory [(Figure 18a](#bookmark107)), with an induction motor. The specimens were placed in the appropriate place of the specimen, and with the rotation of the disc, the surfaces were polished (ground). A detail of the process is shown in the [Figure 18b](#bookmark107)) and the detail of the result in the specimen in the [Figure 18c](#bookmark107)).

**Figure 18 - a) Grinding machine; b) Disc polishing specimen; c) Finalized specimen**


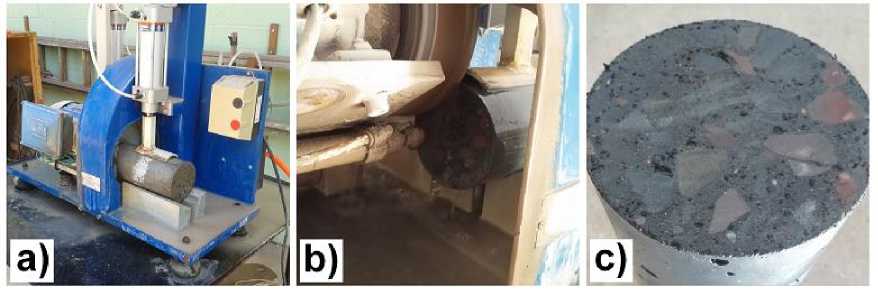


Figure 18 a) Grinding machine; b) Disc polishing specimen; c) Finalized specimen

In this case, as soon as the still-fresh concrete was poured into the mold and vibrated, a paste of cement, water, and superplasticizer was placed on the upper surface. The

The water/cement ratio and the percentage of additives were identical to the trace, which allowed high fluidity due to the absence of dry materials (sand, gravel, rubber, and ash). After 24 hours, the specimens were deformed. The folder used is shown in the [Figure 19a](#bookmark111)). The [Figure 19b](#bookmark111)) shows the cemented specimen compared to another that is still undergoing a rectification process, while the [Figure 19c](#bookmark111)) shows in detail, the thickness of the cementation.

**Figure 19 - a) Paste used in cementation; b) After cementation specimens; c) Detail of cementation thickness**


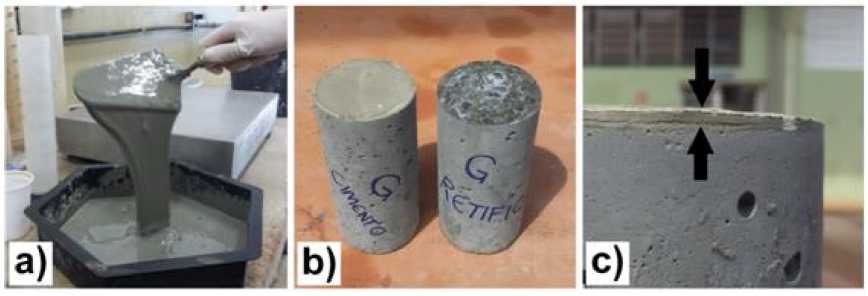


Figure 19 a) Paste used in cementation; b) After cementation specimens; c) Detail of cementation thickness

It was similar to the plywood boards concerning the need for treatment processes in the specimens and how to use them. That is, the plates [(Figure 20a](#bookmark116)) with a size of 11 cm x 11 cm (two on each side, 0.4 cm thick each) were placed at the time of the test, as shown inFigure 20(b)


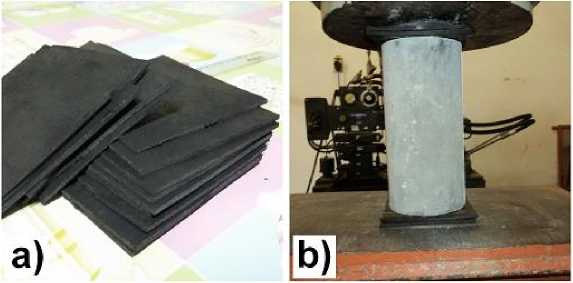


Figure 20 a) Rubber plates; b) Specimen during rubber test
